# Meaning and barriers to quality care service provision in Child and Adolescent Mental Health Services: Qualitative study of stakeholder perspectives

**DOI:** 10.1186/s12913-017-2080-z

**Published:** 2017-02-20

**Authors:** Nadzeya Svirydzenka, Pablo Ronzoni, Nisha Dogra

**Affiliations:** 1 De Montfort University, Leicester, UK; 2 Together NHS Foundation Trust, Hereford, UK; 3 University of Leicester, Leicester, UK

**Keywords:** Mental health, Health care, Quality, CAMHS, Stakeholders, Qualitative

## Abstract

**Background:**

Defining quality in health presents many challenges. The Institute of Medicine (IOM) defined quality clinical care as care that is equitable, timely, safe, efficient, effective and patient centred. However, it is not clear how different stakeholders within a child and adolescent mental health service (CAMHS) understand and/or apply this framework. This project aims to identify key stakeholders“ understanding of the meaning of quality in the context of CAMHS.

**Method:**

The study sample comprised of three groups: (i) patients and carers, (ii) CAMHS clinical staff, and (iii) commissioners (Total *N* = 24). Semi-structured interviews were used to collect data and thematic analysis was applied to explore participant’s views on the meaning and measurement of quality and how these might reflect the IOM indicators and their relevance in CAMHS.

**Results:**

An initial barrier to implementing quality care in CAMHS was the difficulty and limited agreement in defining the meaning of quality care, its measurement and implementation for all participants. Clinical staff defined quality as personal values, a set of practical rules, or clinical discharge rates; while patients suggested being more involved in the decision-making process. Commissioners, while supportive of adequate safeguarding and patient satisfaction procedures, did not explicitly link their view on quality to commissioning guidelines. Identifying practical barriers to implementing quality care was easier for all interviewees and common themes included: lack of meaningful measures, recourses, accountability, and training. All interviewees considered the IOM six markers as comprehensive and relevant to CAMHS.

**Conclusions:**

No respondent individually or within one stakeholder group identified more than a few of the indicators or barriers of a quality CAMHS service. However, the composite responses of the respondents enable us to develop a more complete picture of how to improve quality care in practice and guide future research in the area.

**Electronic supplementary material:**

The online version of this article (doi:10.1186/s12913-017-2080-z) contains supplementary material, which is available to authorized users.

## Background

Providing quality care is the legal and ethical requirement of any healthcare service. Research has identified challenges to achieving quality care include balancing different perspectives, defining accountability, establishing criteria, identifying reporting requirements, minimizing conflict between financial and quality goals, and developing information systems [1]. While clear in the ultimate goal to improve the service, these barriers are not specific or with immediate practical implications. It is necessary to identify practical issues in providing and assessing quality healthcare before meaningful changes can be implemented at service level.

For many years, what constitutes quality of care as well as how to best measure quality have been the subjects of extensive debate by healthcare organisations, politicians, clinicians, hospital managers and patients’ organisations [2–4]. In England and Wales, the National Institute for Health and Care Excellence (NICE) has the role of developing quality standards, in collaboration with health and social care professionals, their partners and service users. NICE is, however, disease specific and focuses on cost-effectiveness for the development of guidelines [5].

Additionally, two other organisations in England play an important role in setting quality standards. Firstly, from 1st October 2010 the Care Quality Commission (CQC) is legally responsible for making sure it meets essential standards of quality and safety [6]. These standards propose: (i) patient involvement in the decision-making process of their care and treatment; (ii) safe and individualised treatment and support to meet the specific needs of the patient to make a difference in their health and wellbeing; (iii) qualified and competent staff to provide the prescribed care in an appropriate and safe environment; and (iv) the service provider to routinely assess the quality of provided services. Secondly, from April 2013, clinical commissioning groups (CCGs) were introduced to replace primary care trusts and have a legal duty to support quality improvement. In particular, they are expected to use outcomes to show: (i) the effectiveness of services, (ii) the safety of services, and (iii) the quality of the patient experience [7].

Finally, international agencies have also advocated for quality frameworks in defining quality healthcare. Most quality indicators focused on the 5Ds models – death, disease, disability, discomfort, and dissatisfaction [8], which some argued focused primarily on the negative aspects of health and service provision ignoring the positive attributes of quality care for health outcomes and daily functioning [9]. Other frameworks focused on three dimensions of care: structure, process, and outcome of healthcare provision or positive health outcomes as a result of quality standards being met [10].

However, the essence of what quality means is still unclear. There is also limited information on how it can be practically achieved at service level with effective clarity for both clinicians and patients. The Institute of Medicine [11] defined quality care as “the degree to which health services for individuals and populations increase the likelihood of desired health outcomes and are consistent with current professional knowledge”. The Agency for Healthcare Research and Quality (AHRQ) summarised the IOM parameters as “doing the right thing, at the right time, in the right way, for the right person – and having the best possible result” [12]. The IOM specified six indicators of quality standards as:

- **Safe**: Treatment helps patients and does not cause harm.
- **Effective**: Where possible the treatment is evidence based
- **Patient**-**centered**: Patients are treated with respect and health professionals need to take account each patient's values about health and quality of life and being responsive to individual variances and needs.
- **Timely**: Patients get the care they need at a time when it will do the most good.
- **Efficient**: Treatment does not waste resources.
- **Equitable**: Everyone is entitled to high quality healthcare irrespective of individual patient characteristics.
  Adapted from IOM, 2001 [13]

The IOM definition and domains are therefore useful in guiding evaluation of care standards in a health care organisation. However, while theoretical frameworks and practical features of quality applicable to service providers may be identifiable, defining barriers to their implementation and measures of quality have been more elusive. This is partly related to the lack of consistent evaluation of quality of health care from the different aspects of care and by all stakeholders involved in the process of delivering or receiving health care [14, 15]. It is especially important that theoretical framework and standards of quality care are for other stakeholders such as patients and commissioners.

Carson and colleagues reported that whereas professionals view quality as based on treatment efficacy and appropriateness, treatment availability, timeliness, continuity, safety, and efficiency; patients assess quality of healthcare based on their impressions of caring, professionalism, competence, and organisation [16]. Similarly, Stichler and Weiss suggest that while providers define quality in terms of patient outcomes, professional standards of practice, predetermined criteria used to measure quality, and even subjective opinion; patients describe it based on the interpersonal aspects of care, how well they were treated and the responsiveness of the provider to their needs [17].

The ability of Child and Adolescent Mental Health Services (CAMHS) to provide high quality care could be improved if we had a clearer understanding of barriers to quality care from the various stakeholder perspectives. This project aimed to explore stakeholder’s views on quality child and mental health service provision and their expectations from these services and how better quality can be achieved. Specifically, this project aimed to identify key issues in CAMHS services from the perspective of patients and families, clinicians, and commissioners. The study employs the IOM theoretical framework to support participants in their understanding of quality by providing a common unified definition and practical domains for reflection. We chose the IOM definition, as it provides specific concepts that allow for a wider understanding of the meaning of quality.

## Methods

This study employed a qualitative cross-sectional design that followed COREQ guidelines for conducting qualitative research [18].

### Sample

We conducted 24 semi-structured interviews with participants from three stakeholder groups: (i) patients (3 interviews with children/adolescents and 4 – with parents/carers); (ii) treatment providers (15 interviews with CAMHS clinical staff including team leaders, managers, consultants, nurses, trainees, and primary mental health workers), and (iii) NHS commissioners who are responsible to identifying and financially supporting CAMHS services that are made available to the public [19] (2 interviews). We followed purposive sampling strategy in identifying the stakeholder groups in line with Ovretveit’s definition of quality, which comprises three distinct dimensions: client quality (what clients/patients and carers *want* from the service), professional quality (whether the service meets *needs* and correctly carries out techniques and procedures), and management quality (efficient and productive use of resources) [20].

NRES NHS ethics committee approved the study. Families were recruited through existing CAMHS database of recently discharged cases. Clinical staff and commissioners were recruited from CAMHS, which is part of a NHS mental health Trust. Initial letters were sent out with the details of the study extending an invitation to participate. After a week, these letters were followed by with a phone call during which desire to participate was assessed and interview was scheduled. After written consent was given, child and parent interviews were conducted separately.

### Data collection

Individual face-to-face audio-recorded semi-structured interviews were conducted with all participants between March and July 2013 by on of the two researchers on the team (please refer to the interview guide in Additional file 1). Location varied by participant group. CAMHS staff and commissioners were interviewed at mutually convenient locations. Children/adolescents and their parents were interviewed separately in a private room in their home. Interview schedule focused on three key areas: (i) the meaning of quality in CAMHS, (ii) quality domains and IOM framework, and (iii) barriers to implementation of quality (child participants were not inquired about quality assessment measures). Saturation in some groups could not be achieved (e.g. commissioners) due to the limited number of particular posts within the area, however the data collected was adequate for the study [21, 22]. Furthermore, low engagement from past patients also limited saturation within that stakeholder group. Saturation in the CAMHS staff group was reached.

## Analysis

Interviews were transcribed verbatim and the data was analysed by multiple members of the team using thematic analysis to identify salient themes and ensure inter-coder reliability. As a research team we were aware of the need for reflexivity [23]. The research team ensured that it identified its own perspectives and assumptions about how the interviewees might understand quality. We also identified themes independently to ensure we countered for possible bias on our parts. Transcripts from each strategic stakeholder group were coded in a three-level coding process [24]. Specifically, first the researcher developed a coding framework that was then further collapsed into 12 themes according to the emerging patterns [24]. These primary themes were then collapsed into three identified meaningful superordinate themes.

## Results

Results of the study are presented across three themes (Table 1): (i) conflicts in quality definition, (ii) conflicts in IOM framework interpretation; (iii) other barriers to quality implementation. Direct quotes are used and we identify the source of the quote but use generic descriptors to preserve anonymity.

**Table 1:** Outline of themes and sub-themes identified across three key stakeholder group interviews

*Theme*	Stakeholder group		
	CAMHS Staff	CAMHS commissioners	CAMHS patient groups
*1: Conflicts in quality definition*	- Fluid and nebulous - Reflects variety of stakeholder opinions - Based on policy, theory, & research evidence - Dynamic in integration and execution of different perspectives - Personal qualities and values of staff - Paramount in assessment and treatment	- Seen as patient experience, outcomes, and safe-guarding - Management of patient queries/complaints - Paramount in meeting patient expectations	- Reputable and recommended service - Knowledgeable, trustworthy, and communicable staff - Attention to the individual needs of patients - Prioritised staff qualities over facilities - Paramount in access to services and timely treatment
*2: Conflicts in IOM framework interpretation*	- All were seen as relevant - Timeliness and patient-centeredness: pros & cons due to individual circumstances - Suggested three more domains: sustainability, staff wellbeing, and multiagency working	- All were seen as relevant - Emphasised patient safety issues and timeliness of offered services - Suggested multiagency working as holistic approach to meeting mental health needs of patients	- All were seen as relevant. - Parents prioritised timeliness and efficiency - Young patients felt that all six were represented in the treatment they received
*3: Barriers to implementation of quality care in CAMHS*	- Identified issues with existing measures of quality - Suggested improved ways to measure quality - Identified a wide range of barriers at service level to defining, measuring, and delivering quality services	- Addressed existent issues in reports on guidance and medication - Either identified broad national measures of quality were not aware of them - Could not identify outcomes of known measures - Were in consensus that quality needs to be measured - Identified the need to be better informed through concise reports and improved communication with all levels - Suggested change in how services respond to GPs/commission	- Suggested alleviating first-appointment anxieties - Identified the need for child-friendly communication letters - Wished for increased access (referral, timing) - Asked for a choice of clinicians *Patients were not asked about existing measures of quality as it was not applicable to their experience of CAMHS*

### Theme 1: Conflicts in quality definition

One of the fundamental issues in implementing quality care at CAMHS is the lack of a consistent definition of what quality actually means across different stakeholder groups. Most CAMHS staff viewed providing quality as ‘*the most important part of my role’ (Child and Adolescent Consultant Psychiatrist: CAP)* as well as being applicable to all aspects of the job *‘all my roles should involve quality’ (Specialist nurse: SN).* The particular meaning of the word ‘quality’ was challenging to define as *‘quality is a very nebulous term and the interpretation of quality is different for different individuals’ (CAP)* was a popular opinion.

Interviewees valued a definition of quality as one that took into account all stakeholder perspectives, and had an underlying theoretical model of mental health practice based on clinical expertise and policy guidelines:

*‘… what the patient and the carers feel comfortable with, what their expectations are. … [it is] interaction with that professional, rather than to do with the buildings’ (CAP)* although the state of the building may impact on safe care

Furthermore, the harmonious relationship between these levels was seen as necessary:

‘*quality is a good formulation, good liaison with the professionals involved that being pulled together and co-ordinated’ (Team Lead)*

From patient perspectives, quality of care primarily comprised of reputable and trustworthy services; interaction between patient and clinician, security and confidentiality (later seen in the IOM domains). CAMHS attention to key stages of transition for their patients (e.g. from child to adult services) and continuous support for the case were also seen as indicators of quality.

‘*They are very kind to you and treat you separately to others [anonymously] and making sure that you’re looked after properly.’ (Patient aged twelve)*

‘*I could really express myself to someone and not be judged for it. And I found that really helpful, cos I think it’s quite rare that with mental health to be able to talk to someone that openly and not expect oh god what a complete weirdo.’ (Patient aged eighteen).*

Quality for CAMHS commissioners meant ‘*addressing patient expectations*’ and ‘*appropriate investigation of identified issues’*. They expressed that they ‘*are driven by what the service users expect and so they try to commission with their expectations in mind’*.

### Theme 2: Conflicts in IOM framework interpretation

Introducing IOM framework in the interview was a welcome reference by all participants. Most commented how they had already mentioned some of the identified six domains earlier in their own definition. However, no one mentioned all six domains prior to the prompt being introduced. Commissioners viewed the domains as a useful frame work to incorporate in conceptualising quality care in CAMHS, but struggled to elaborate as to how this might be applied. All patient and parent interviewees felt that the six domains were appropriate and reflected their experience. Furthermore, interviews revealed discrepancies of how different domains of quality were being prioritised by different stakeholders.

#### Safe

Safety of CAMHS services comprised of: (i) risk assessment and no harm being caused, (ii) competency of staff, and (iii) staff accountability. While patients agreed that the place was safe, staff at CAMHS emphasised the importance of their role in facilitating the sense of security in patients and their family.

*‘Safety has to come first. If I’m working with a troubled teenager who is putting themselves in vulnerable situations, one of the things I would be focusing on is doing a risk assessment and reminding them, look you have to keep safe and it is my job to help you keep safe.’ (CAP)*

*‘The place was good, didn’t have to worry about anyone else walking in, I think the structure of it was good they didn’t push you to do too much in one session.’ (Patient aged fifteen)*

#### Effective

Effective care was seen by all respondents as evidence-based practice (e.g. NICE guidelines), with sufficient clinical expertise and the specific nature of clinical therapy.

*‘Effective is … probably the most obvious one… symptomatic change although whether you can always attribute change to the intervention is very difficult in mental health. (CAP)*

*‘She [clinician] was absolutely on the ball and knew exactly what she was doing and what Mark was doing.’ (Parent)*

#### Timely

Timely was a controversial domain because although the majority of clinical staff agreed that care should be received with minimal delay to be most effective, they felt *‘willingness to engage with therapy’ (Psychotherapist)* on the part of patients and staffing issues, were worth considering. Parents were primarily concerned with the timeliness of referral and care received as well as whether it has had an effect on the child and alleviated the symptoms.

*‘that’s where the problem is: it takes an awful long time to know what’s gonna happen’ (Parent)*

Commissioners agreed on the importance of timeliness and emphasised making it measurable to address patient expectations.

#### Patient centred

While clinical staff were unanimous in the need to identity the patient’s opinion and being patient-centred, they were cautious in this being the sole determinant in influencing treatment choices. They advocated transparency and collaboration with patients but highlighted the need for clinical expertise in leading the treatment.

*‘Do patients, service users, families whoever feel that we’ve made a difference in the areas we can make a difference in’ (Clinical Psychologist)*

*‘…if you just stay with […] what the patient wants […] I think it might take away the element of quality for what I consider which is to be able to offer better insight and understanding for the patient for what they are experiencing.’ (Trainee Psychotherapist)*

*‘Listening to the patient I think… that certainly happened from our point of view’ (Parent)*

#### Efficient

Efficient care has generally been interpreted as value for money that is a good use of resources.

*‘when people are talking about efficiency it’s so financially driven that it’s often a word that’s used to justify a lack of quality … we need to be efficient but there’s got to be a line where you say that actually it is starting to impact on quality now and we can’t make any more cuts.’ (Clinical Psychologist: CP)*

#### Equitable

While clinical staff were consistent in their views that patients at CAMHS receive equitable care, they were concerned that where patients lived affected access.

*‘as individuals we would treat everybody according to their health needs […] but, just in terms of where they live, they may or may not get something…’ (Specialist Clinical Director)*

*‘… having a service that is culturally appropriate and sensitive to the needs of different communities … is really effective.’ (CAMHS commissioner)*

### Theme 3: Practical barriers to quality implementation and possible solutions

In spite of their sometimes different views on what quality is, CAMHS staff and commissioners were forthcoming about the barriers to implementing quality care consistently and sustainably.

*‘there’s a lack of understanding of what CAMHS is about from above. … On one hand you’re having the demands of you need to cut this much money and how do you do that without effecting quality care and then also improving on it.’ (Community Psychiatric Nurse: CPN)*

*‘We don’t get a lot of feedback … we just get some generalised bar charts…’ (CP)*

CAMHS staff group identified ten practical barriers with some of these being identified by individuals and others by several interviewees:

#### Poor existing quality measures

While clinicians and commissioners recognised the importance of having a practical definition of quality and the usefulness of the IOM framework and domains in clinical care (albeit focus being different for some stakeholder groups), their adequate and meaningful measurement posed more of a challenge. There was consensus for better, clearer, more applied, clinically relevant, and consistent measures of quality with relevance to all stakeholders. Achieving this in the current climate and existing organisational structures was considered to be difficult.

*‘Thinking about it most clinicians are aware of those components of quality. But the way it’s measured in CAMHS is probably not objectified enough, it’s not measurable enough.’ (Trainee Child Psychiatrist: TCP)*

Apart from routine data about case numbers, interventions, and flagging up cases that were breaching waiting times or routine questionnaire measures part of the national initiative, commissioners were not aware of any further quality measures. They suggested user feedback and short and long-term outcome-based assessments of patient satisfaction, multiagency working, and centralising service management. Needless to say, lack of consensus on what measures need to be implemented and how to sustainably and reliably to measure quality is one of the key barriers to improving quality care in CAMHS.

#### Poor sustainability of measures

Interviewees stressed the importance of quality as an ongoing matter in all aspects of service provision.

*‘I would add a seventh which would be something to do with what can make the process sustainable. Because it’s important that quality is not something that you do in bursts … There could be a risk that the quality will not be sustainable.’ (CAP)*

#### Target- instead of quality-driven services

Identifying clinically-meaningful quality parameters was seen as key in implementation of good quality practice.

*when it’s [quality] very target driven I think actually quality goes down rather then up so what we’re measuring isn’t quality, what we’re measuring is staff compliance to targets which I don’t’ think is particularly helpful (CP)*

However, current systems were not supportive of the quality instead of target focus.

#### Job apathy and lack mutual accountability

CAMHS staff advocated for more intra-team accountability:

*‘When many people work together it helps in a way to ensure quality because if somebody is drifting off course, it brings us in line. Equally in our team quality is noted by peer review in the way we conduct ourselves and from triage to meetings and our writing and so on’ (CP).*

A close collaboration with other agencies involved in the care of that child were integral to quality service provision.

*‘We’ve got different agencies involved in a young person’s care; agencies like education, social care, CAMHS, paediatrics, general hospital…’ (TCP)*

However, these relationships can be challenging as in trying to fill the provision gaps in other services may mean that the service may not effectively perform its primary function well.

#### Communication and governance

Clear lines of communication need to be established between all stakeholders: between patients and clinicians, between Trust and CAMHS, within clinical team.

*‘there’s things that happen above that are not within our control … there’s a lack of understanding of what CAMHS is about from above. … On one hand you’re having the demands of you need to cut this much money and how do you do that without effecting quality care and then also improving on it and when’ (CPN)*

In response, some offered focusing on patient needs, identifying what those are, and the methodologies that might be useful in doing that:

*But more qualitative feedback from young people, I guess that’s something that we’re not that great at really… So I do feel that when we get groups of young people together, we are very good at listening to them, but it’s, it’s just having time to do that I think. (Primary mental health worker)*

Patients’ suggestions centred on communication, such as offering introductory leaflets to ease anxiety before appointments and reassurance of staff’s trustworthiness. Using child-friendly language in letters to ease comprehension by patients without resorting to parents for interpretation was also suggested.

*‘before I started I was like really like anxious and I would have loved to have known, like had somebody tell me that … “don’t like be anxious, don’t be worried, cos it’s like, they’re really good and they will definitely like help you out” … give out sort of like patient story kind of like leaflet things or something.’ (Patient aged eighteen)*

#### Lack of guidelines and training related to quality service provision

*‘lots of policies and procedures have been rushed through the Trust systems in the last eighteen months and there’s been a significant lapse of training in some of those policies, but expecting staff to operate them and have a working knowledge of them to maintain the quality and safety but the Trust hasn’t been able to provide that training.’ (Specialist Clinical Director)*

CAMHS staff recognised not only the need to offer basic care, but to improve and train staff to be able to support the demand on the service and provide best evidence-based treatments. If opportunities for training were not created, this was said not only to affect the treatment of patients, but also the well-being of staff.

*‘if people genuinely want to give a high quality service, but feel they can’t, they get very stressed, anxious, unhappy because they feel like they’re being squeezed and they can’t deal with that.’ (SN)*

#### Lack of analysis of and feedback from quality measures or justifiable baseline standards for interpretation of findings

Several clinicians commented that even when quality measures are administered, there is clear lack of strategic analysis and feedback from the data collected.

*We don’t get a lot of feedback … if you are just collecting information and then it goes some where I do not know and we just get some generalised bar charts, where has my individual bit and feedback really. (CP)*

#### Bureaucracy and access

CAMHS staff while recognised the need to measure quality commented on the bureaucratic nature of the task thus undermining the possible useful outcomes in performing such tasks.

*It’s [measuring quality] just seen as another admin task really… (CP)*

On the flip side, parents wished for easier access to services and less bureaucracy in the process. Referrals and appointments generally require lengthy paperwork and therefore timing does not match the urgency of the need.

*‘it doesn’t seem caring if you can’t access it when… and if you need to …’ (Parent 1)*

*‘the referral into CAMHS was eventually done in, that process took a long time […] it takes an awful long time to get to know what’s gonna happen’ (Parent 2)*

Commissioners acknowledged the desire to stay better informed about quality care in services through reports as well as evidence of change in response to GP concerns. They were not clear about the practical changes that CAMHS might make to minimise associated bureaucracy.

#### Values and team morale

CAMHS staff emphasised when discussing quality care and IOM domains that they represent concepts deeper than a theoretical framework. Interviewees stressed that achieving quality care as defined in the IOM domains CAMHS as a whole and employees specifically need to internalise these values. Therefore, quality provision might not be achieved if those values are not individually and collectively endorsed.

*‘A lot of these [quality domains] are value embedded aren’t they, that’s what’s coming across as we’re talking about it. And we have a go at measuring some of them but a lot of it’s about, seems to me to be about how you work as a group of people, how you work, the values you hold, the values you construct between you. How you see yourselves as a team.’ (SN)*

*‘I think that to motivate members of the team, it helps [to provide quality care] if they are, if they feel they are doing something worthwhile, something, which is really useful for the young persons and the family.’ (CAP)*

#### Lack of resources

It is clear from individual and collective account of all interviewees that quality suffers with limited resources. Lack of resources means that quality itself is not directly and systematically assessed:

*In an ideal world, I will that we had more systematic quality control but it has to be given the allocated resources it deserves. … I feel that there should be a nominated person in the team whose job is to monitor the quality that we do … a researcher embedded in the team (CAP)*

However, secondary to quality assessment, provision of care is also heavily dependent on resources, the lack of which undermines the extent to which the six key domains of the IOM framework can be implemented within CAMHS. Importantly, the pressure on staff to assess and treat with limited resources, to do things quickly, peer support networks staff health, stress management, and staff capacity were all seen as factors that can impact on the quality of care provided, but are not explicitly part of the IOM framework.

*‘… currently there is a lot of stress amongst us. And that’s very important when it comes to quality of the service, if I am stressed, I’m not doing the best thing that I can to my patient, isn’t it?’ (CP)*

Parents also recognised capacity issues but wanted choice of clinicians (especially in gender sensitive cases) and increased funding to improve capacity and access.

*‘… I’d like you to have lots more funding and plenty more staff and to be able to help anybody who is referred’ (Parent)*

Essentially quality service came down to doing the most with what was available:

*‘we can deliver the best service in the world but if it’s not affordable, no-one’s going to get it’ (SN).*

## Discussion

In-depth interviews with targeted groups of stakeholders in CAMHS showed that while commitment to quality is unanimous and strong, barriers to sustainable and transparent quality delivery and assessment can be diverse. While all the stakeholders found the IOM framework helpful it appears to be hindered by the subjective nature of some of the components’ factors, as perspectives on what quality is varies with the role and experience of the stakeholder, be it patient, clinician or commissioner. Further work needs to focus not only on naming domains of quality of healthcare, but also facilitating common understanding of what these domains mean, in quality terms, to all stakeholders. More specifically, whereas parents/carers and children/adolescents emphasised timeliness and personable staff as quality indicators respectively; commissioners, in line with Overetveit’s previous reports, focused on complaint processes suggesting that they tend to rely on the customer service domain [20].

Diverse perspectives on defining quality care came from CAMHS clinicians. They highlighted aspects already reported by Carson and colleagues in other healthcare settings regarding treatment efficacy and appropriateness, treatment availability, timeliness, safety, and efficiency; and also emphasised empathy, warmth, teamwork and listening skills [16]. These clinicians appear to give equal value to the human qualities of delivering healthcare as to technical skills.

NICE was the most cited policy document in guiding quality care in CAMHS among clinicians. However, commissioners made no direct reference to the framework guiding their understanding of quality. They veered on the side of selecting correct treatment, improving psychological symptoms, and discharging the patient in efficient and timely manner. Furthermore, they emphasised patient-led treatment meaning treatment that meets the expectations of the patients. This may be a somewhat simplistic view as patients may expect a diagnosis but if diagnostic criteria are not met, irrespective of their wishes it would be clinically inappropriate to give them a diagnosis albeit that it what they want. Perhaps understandably, neither patients nor carers seemed aware of frameworks to define quality of care. NICE may need to provide a more comprehensive description of what a high-quality service should look like, making it more available and accessible to the general public.

The number of barriers identified by the participants in delivering and measuring quality care in CAMHS raises concerns. Our findings concur with those described by McGlynn [1]. Clinicians highlighted the need for transparency on what should be measured and how this can be measured. This approach would enable consistency and may increase the likelihood of success. Furthermore, the need to have a clear conceptualisation of terms before trying to measure quality is vital.

Three key routes to supporting quality care in CAMHS taking into account the current restraints are suggested. The first is to develop a framework from both policy and clinicians’ perspective in consultation with patients. CAMHS staff appeared critical of the current single top-down perspective on quality care as they argued it missed clinical expertise and application.

Second, CAMHS staff argued for clinically valid measures of different quality domains. These measures need to overcome the barriers identified by clinicians so should not be labour intensive quantitative measures of outcomes were deemed good, the general consensus proposed qualitative measures in view of peer reflection, supervision practice, and active engagement with users could also measure quality. Although they accepted it is a challenge, CAMHS staff argued in favour of a single measure that is meaningful to all stakeholders and is integrated into all aspects of service planning and practice.

Finally, for improved communication, patients suggested using more child-friendly language in correspondence and offering brochures prior to first appointments which could include testimonies from former patients to alleviate anxiety. A system of measuring quality that is clinically valid is vital. The end result would be the development of research inspired by clinical practice and clinical practice informed by research findings. The only way to achieve this is to have a framework developed from both policy, user, and clinician’s perspective, as the current single top-down perspective misses clinical expertise and application.

Although this study has provided some understanding of stakeholder perspectives of the existing barriers to quality health care in CAMHS and possible solutions, there are several limitations. The qualitative design restricts a broad generalisation of the findings. However, the CAMHS service investigated is likely to be similar to many others across the UK. Our findings could complement a more comprehensive review of quality standards in CAMHS across the UK, to which end the model of the study we present in this paper is a useful guideline. The commissioner perspective is perhaps the most limited as the number was small and commissioners are less likely to share a professional background so generalising this perspective is less possible. Small number of commissioner interviews is a clear limitation of the current study, as their insight would have allowed for deeper understanding of how specialist services like CAMHS are governed. As commissioners are charged with the task of determining which health care services to prioritise and fund and therefore to make available to the local population, quality of CAMHS services and the variety of needs they can address can therefore be seen as subject to this top-down commissioning process. It would be helpful in developing a functioning quality framework to include discussions with higher levels of NHS Trust governance, which was only possible to a very limited extent in the current study.

## Conclusions

This study identified how the IOM domains can be applied to achieve consistent good quality care. The IOM focus is rightfully on the patients, and our study shows that all stakeholders agree that for patients to receive good quality care it needs to be equitable, timely, safe, efficient, effective and patient centred. However, IOM is missing an important aspect; that is to provide good quality care, clinicians, nurses, and other CAMHS staff need to be supported, valued, rested, and motivated amongst other things. Therefore, IOM framework fails if CAMHS services are not evaluated as a complex interdependent mechanism that they are.

While IOM is useful in thinking about quality of CAMHS services once the child has been referred, there is also a clear need to expand the scope and develop suitable means of addressing quality of providing equity in reaching specialist services. Understanding cultural, ethnic, and economic factors that shape the needs and build access barriers in local population is one avenue. Another – is addressing systematic hierarchical and bureaucratic difficulties inherent to the NHS system highlighted in our study in reaching disadvantaged populations. Making use of epidemiological research [25] and latest technologies could offer the necessary insight and possible solutions to meeting the needs of underprivileged populations.

The study also highlighted the need to better define the meaning of terms currently used to define quality, as although there was agreement in the domains, what they really meant differed considerably between the stakeholders. It appears that CAMHS staff views are closer to service users’ views but further away from commissioners. The lack of commonality in the understanding of what quality of health care in CAMHS means for different stakeholders as well as systematic resolution of existing barriers and stakeholder concerns appears to hinder the development of a successful framework to understand and implement quality consistently across the UK.

## Additional file

Additional file 1: Interview guide – Stakeholder interviews (all groups) – Qualitative interview data. (DOCX 22 kb)

## Abbreviations

AHRQ: Agency for healthcare research and quality
CAMHS: Child and adolescent mental health service
CAP: Child and adolescent consultant psychiatrist
CCGs: Clinical commissioning groups
CP: Clinical psychologist
CPN: Community psychiatric nurse
CQC: Care quality commission
IOM: The institute of medicine
NHS: National health service
NICE: National institute for health and care excellence
SN: Specialist nurse
TCP: Trainee child psychiatrist
