# Supplementary material for: Meaning and barriers to quality care service provision in Child and Adolescent Mental Health Services: Qualitative study of stakeholder perspectives
Source: BMC Health Serv Res. 2017 Feb 20;17:151. doi: 10.1186/s12913-017-2080-z (PMC5319051; doi:10.1186/s12913-017-2080-z)
Supplement: Additional file 1: — Interview guide – Stakeholder interviews (all groups) – Qualitative interview data. (DOCX 22 kb) [file 12913_2017_2080_MOESM1_ESM.docx]

**INTERVIEW GUIDE**

**Research title:** Exploration of meaning and barriers to quality in Child and Adolescent Mental Health Services

**Interview Topics & Prompts for Children**

- **CAMHS experience**: I wonder if you can tell me what you know about CAMHS? Please, tell me about your experience with CAMHS? What was good or positive about the contact? What was not so good or negative about the contact?
- **Quality definition**: What do you think the word quality means? Can you give an example of something that is good quality? How do you think we can decide if something is of good quality or not? How do you think we might apply that kind of thinking to CAMHS? In terms of quality what was good about CAMHS? Can you give me an example to explain more about that? What was not so good about CAMHS? Why? Can you give an example?
- **Quality improvement**: Thinking back on your time at CAMHS, what would you like to have happened differently? What would you like to change? Do you think services can always make the changes suggested? What kinds of things might stop services improving?
- **Quality domains**: A big organisation in the United States called the Institute of Medicine came up with some words to describe the way they saw quality care. (Provide card with domains). Do you think the care you received while at CAMHS was like the care the IOM is describing? What do you think care that is safe, effective, patient centred, efficient and equitable looks like?
- **Reflection & Conclusion:** Have your views changes at all about CAMHS from the first time you encountered CAMHS and after you left the services? What would you like to add to what we’ve just discussed?

The card with domains will say:

- Safe – meaning no harm is caused either physically or mentally
- Effective – the intervention helps/ makes things better
- Timely – the care is given at the right time depending on the patient’s needs
- Patient centred – the views of the patient are taken into account when planning care
- Efficient – there is a good use of whatever resources are available
- Equitable – patients get the care they need regardless of their background

**Interview Topics & Prompts for Parents**

- **CAMHS experience**: Can you tell me about your first experience with CAMHS? How did it make you feel?
- **Quality definition**: What do you think the word quality means when applies to mental health services? How would you evaluate your visit to CAMHS in Leicester in terms of the quality? What was good about your experience with CAMHS? What was bad about your experience with CAMHS? Why? Can you give an example?
- **Quality improvement**: Thinking back on your time at CAMHS, what would you like to have happened? What were your expectations in terms of quality care you were to receive? Having completed your treatment at CAMHS, what would you like to change? What would you would like to see be added that you think other families like your own might find useful?
- **Quality domains**: What would you list as good quality factors of care provided by CAMHS? Can you describe how can service be safe, effective, patient-cantered, timely, efficient, and equitable? (Provide card with domains). Do you think the care you received while at CAMHS was like that?
- **Reflection & Conclusion:** Have your views changes at all about quality care CAMHS from the first time you encountered CAMHS and after you left the services? What would you like to add to what we’ve just discussed?

**Interview Topics & Prompts for CAMHS Staff**

- **CAMHS experience**: Can you please describe your role with CAMHS?
- **Quality definition**: What do you think the word quality means when applies to mental health services?
- **Quality measurement**: Do you use any systematic ways of assessing quality at CAMHS? If yes, which? If no, why not? What are your thoughts on existing measures of quality? How would you choose to assess the level of quality care at CAMHS?
- **Quality domains**: How would you describe good quality care in child and adolescent mental health services? What key domains would you identify? Can you give an example of each domain you just listed? Institute of Medicine have suggested six domains of their own: safe, effective, patient-cantered, timely, efficient, and equitable? (Provide card with domains). What are your views on these domains?
- **Conclusion:** What would you like to add to what we’ve just discussed?

**Interview Topics & Prompts for NHS Commissioners**

- **CAMHS experience**: Can you please describe your role with CAMHS?
- **Quality definition**: What do you think the word quality means when applies to mental health services?
- **Quality measurement**: Do you use any systematic ways of assessing quality at CAMHS? If yes, which? If no, why not? What are your thoughts on existing measures of quality? How would you choose to assess the level of quality care at CAMHS?
- **Quality domains**: How would you describe good quality care in child and adolescent mental health services? What key domains would you identify? Can you give an example of each domain you just listed? Institute of Medicine have suggested six domains of their own: safe, effective, patient-cantered, timely, efficient, and equitable? (Provide card with domains). What are your views on these domains?
- **Conclusion:** What would you like to add to what we’ve just discussed?
